# Glycemic status and health-related quality of life (HRQOL) in populations at risk of diabetes in two Latin American cities

**DOI:** 10.1007/s11136-023-03398-x

**Published:** 2023-04-03

**Authors:** Luis A. Anillo Arrieta, Karen C. Flórez Lozano, Rafael Tuesca Molina, Tania Acosta Vergara, Sandra Rodríguez Acosta, Pablo Aschner, Yenifer Diaz Montes, Julieth P. Nieto Castillo, Víctor Alfonso Florez-Garcia, Noël C. Barengo

**Affiliations:** 1 Division of Basic Sciences, Department of Mathematics and Statistics, Universidad del Norte, Barranquilla, Colombia; 2 Division of Health Sciences, Department of Public Health, Universidad del Norte, Barranquilla, Colombia; 3 ScienceFlows Research Group, University of Valencia, Valencia, Spain; 4 Division of Humanities and Sciences, Division Social, Department of Economics, Universidad del Norte, Barranquilla, Colombia; 5 Asociación Colombiana de Diabetes, Bogotá, Colombia; 6 Javeriana University, Bogotá, Colombia; 7 San Ignacio University Hospital, Bogotá, Colombia; 8 Department of Translational Medicine, Herbert Wertheim College of Medicine, Florida International University, Miami, FL USA; 9 Department of Public Health, Faculty of Medicine, University of Helsinki, Helsinki, Finland; 10 Department of Global Health, Robert Stempel College of Public Health and Social Work, Florida International University, Miami, FL USA; 11 Joseph J. Zilber School of Public Health, University of Wisconsin, Milwaukee, USA

**Keywords:** Health related quality of life, Prediabetic state, Type 2 diabetes, Risk factors, FINDRISC

## Abstract

**Purpose:**

To estimate the health-related quality of life (HRQOL) according to glycemic status, and its relationship with sociodemographic and clinical factors in a population at risk of developing type 2 diabetes (T2D).

**Methods:**

Cross-sectional study, using cluster sampling. Data were collected from 1135 participants over 30 years of age, at risk of developing T2D from the PREDICOL project. Participants' glycemic status was defined using an oral glucose tolerance test (OGTT). Participants were divided into normoglycemic subjects (NGT), prediabetes and diabetics do not know they have diabetes (UT2D). HRQOL was assessed using the EQ-5D-3L questionnaire of the EuroQol group. Logistic regression and Tobit models were used to examine factors associated with EQ-5D scores for each glycemic group.

**Results:**

The mean age of participants was 55.6 ± 12.1 years, 76.4% were female, and one in four participants had prediabetes or unknown diabetes. Participants reported problems most frequently on the dimensions of Pain/Discomfort and Anxiety/Depression in the different glycemic groups. The mean EQ-5D score in NGT was 0.80 (95% CI 0.79–0.81), in prediabetes, 0.81 (95% CI 0.79–0.83), and in participants with UT2D of 0.79 (95% CI 0.76–0.82), respectively. Female sex, older age, city of residence, lower education, receiving treatment for hypertension, and marital status were significantly associated with lower levels of HRQOL in the Tobit regression analysis.

**Conclusions:**

HRQOL of NGT, prediabetes, and UT2D participants was statistically similar. However, factors such as gender, age. and place of residence were found to be significant predictors of HRQOL for each glycemic group.

## Plain English summary

Diabetes is one of the main noncommunicable diseases with an increasing prevalence in the world, which has turned it into a serious public health problem. In Colombia, in 2019, diabetes affected 8.4% of the adult Colombian population and more than one million adult Colombians in this age group have hidden or undetected diabetes. Diabetes is not only characterized by increased premature mortality, loss of productivity, economic impact, but it also entails a deterioration in the quality of life of people with diabetes and their respective families. However, little is known about health-related quality of life (HRQOL) in population with different glycemic states. This study investigated the quality of life in patients with diabetes risk, glucose intolerance, impaired fasting glucose, T2D, and its association with some sociodemographic, lifestyle, and background variables, and established the difference of these in two territories of Colombia. The results of this study indicated that no statistically significant associations were found in HRQOL in the NGT, prediabetes, and UT2D groups. However, some sociodemographic and clinical factors were significant predictors of HRQOL in some glycemic groups. Therefore, more attention should be paid to these determinants of HRQL to design and implement strategies to improve these variables, aiming to reduce the risk of deterioration in the quality of life of prediabetic or diabetic patients.

## Introduction

Type 2 diabetes (T2D) has become one of the fastest growing health emergencies of the twenty-first century. An estimated 537 (10.5%) million adults worldwide have diabetes, and 860 (16.8%) million have prediabetes, i.e., impaired glucose tolerance (IGT) and/or impaired fasting glycemia (IFG). It is expected that, by 2045, the prevalence of diabetes will be 12.2%, while that of prediabetes will reach 17.5% [1, 2]. In Colombia, the prevalence of diabetes and prediabetes is 8.3% and 20.7%, respectively, and it is estimated that at least 1 in 3 diabetics do not know they have diabetes (UT2D) [1]. For these reasons, it is essential to identify people at risk of diabetes, prediabetes or unknown diabetes on a time [3–7].

Multiple studies show that T2D negatively affects HRQOL, due to the chronicity condition, metabolic control, treatments, and complications of the disease [8–18]. However, most of these studies are in population with a previous diagnosis of diabetes, so very little is known about what HRQOL is like in subjects with unknown type 2 diabetes (UT2D). Likewise, there is no clear consensus about HRQOL in prediabetic states, due to the limited and controversial literature on HRQOL in population with prediabetes [19–23]. In addition, most studies assessing HRQOL in populations at risk of diabetes only include participants with glycemic impairment, so there are few studies that include normoglycemic subjects (NGT) who are at risk of developing T2D according to The Finnish Diabetes Risk Score (FINDRISC ≥ 12) [3, 22, 24].

Measurement of HRQOL refers to the subjective assessment of a broad range of dimensions of current functional health that affect overall well-being [25]. There is now a growing interest in including patient-reported outcome measures (PROs) because they capture aspects of treatment effect that may not be captured in the main clinical outcomes [26, 27]. Hence their great importance in the field of chronic diseases or epidemiological studies as an indicator of clinical effectiveness and health economic evaluation [26, 27].

HRQOL is assessed using disease-specific measures or generic instruments such as the Short Form 36 (SF-36) [28], the health utility index (HUI) [29], and the EQ-5D [30], which stands out, initially assessing health status in severity levels by dimensions and then on a visual analog scale (VAS) [31, 32]. In recent scientific literature, the EQ-5D is the most used instrument to assess HRQOL in the T2D population; however, it has been little used in the population at risk for diabetes or prediabetes [33]. In a previous study with the same data set, the determinants of HRQOL were established according to the risk of diabetes [24]. However, that study did not consider establishing the HRQOL for each glycemic status, which is very important if one wishes to calculate quality-adjusted life years (QALYs) in those studies that seek to evaluate the effects of an intervention or the evaluation of a new health technology.

To our knowledge, there is no information Available at studies that establish HRQOL according to glycemic status in population at risk for T2D using the EQ-5D-3L in Colombia or Latin America. Therefore, the objective of our study was to estimate the HRQOL according to glycemic status, and its relationship with sociodemographic and clinical factors in a population at risk of developing T2D.

## Materials and methods

### Study design and sample

A cross-sectional study was designed from the study Evaluation of a community health program to prevent type 2 diabetes and other cardiometabolic risk factors in adults (–PREDICOL-ClinicalTrials.gov identifier NCT03049839). The methodology of this study has been previously published [24]. In summary, 1135 participants were recruited in the PREDICOL clinical trial, developed in neighborhoods with low socioeconomic strata in two Colombian cities between 2018 and 2019. The cities included in this study were Bogotá (large city) and Barranquilla, the first and fourth cities, respectively, with the largest number of inhabitants in the country [34, 35].

### HRQOL assessment

HRQOL was assessed using the generic EQ-5D-3L questionnaire, which comprises five dimensions: Mobility (MO), Self-care (SC), Usual activities (UA), Pain/discomfort (P/D), and Anxiety/depression (A/D). Each dimension consists of three severity levels corresponding to no problem (1), moderate problems (2) and extreme or severe problems (3). The combination of the digits of all dimensions generates a 5-digit number describing the participant's health status, there being 243 possible health states [30, 36]. For example, state (11111) represents that the participant reports no problems in any dimension and (33333) represents extreme or severe problems in all dimensions, while state (11121) indicates moderate pain/discomfort problems and no problems for the other dimensions. Moreover, the participant should mark the point on the vertical line of a 20 cm Visual Analog Scale (VAS) rating of his or her overall health status denoted as worst imaginable health status (0) to best imaginable health status (100) [30].

Because of Colombia does not currently have an EQ-5D-3L value set [37, 38], the EQ-5D score in this study was calculated using the Latin American value set [39] as suggested by recent guidelines from agencies in charge of health economic evaluations [40, 41]. This set has 243 utility values which were obtained by the *time trade off* valuation technique "TTO" where the states (11111) and (33333) represent utility values of 1 and − 0.101, respectively. Negative HRQOL values mean that some health states are considered worse than death.

### Diabetes risk

The risk of T2D was identified and stratified by means of the Finnish Diabetes Risk Score (FINDRISC) questionnaire. The FINDRISC is a questionnaire with eight questions, in which each response is assigned a score, 8 items are considered with categorized responses on Body Mass Index (BMI), age, waist circumference, self-reported physical activity, daily consumption of fruits or vegetables, history of glycemia alteration, and family history of T2D [3, 4]. This instrument has been validated and adapted for various populations worldwide as in different Latin American countries, including Colombia, where the weighting and stratification of the total risk is obtained from the simple sum of the individual weights of each response. The FINDRISC score ranges from 0 (minimum score) to 26 (maximum score) points, and, for Colombia, a FINDRISC score ≥ 12 defines a risk of developing T2D in the next 10 years [5–7].

### Glycemic classification

An oral glucose tolerance test (OGTT) was performed on study participants identified as being at risk for T2D (score ≥ 12 on the FINDRISC questionnaire). According to the recommendations and diagnostic classification criteria issued by the World Health Organization, an initial fasting glycemia was performed, followed by providing the patient with a solution containing 75 g of glucose; where two hours after ingestion of the glucose serum, a second glycemia sample is taken (Glycemia 2 h) [42]. Participants who had a fasting plasma glucose (FPG) level ≥ 126 mg/dl or plasma glucose at 2 h (2hPG) ≥ 200 mg/dl were classified as UDT2. Those with 2hPG ≥ 140 mg/dl, but < 200 mg/dl and FPG < 126 mg/dl were classified as impaired glucose tolerance (IGT). Impaired fasting glucose (IFG) was defined as FPG ≥ 110 but < 126 mg/dl and 2hPG < 140 mg/dl. Participants with FPG ≥ 110 but < 126 mg/dl and 2hPG ≥ 140 mg/dl but < 200 mg/dl were defined as combined IGT and IFG. Finally, participants with values below 110 mg/dl in basal glycemia and 2hPG < 140 mg/dl respectively were classified as normotolerant (NGT). Prediabetes was defined as participants with some abnormal glucose tolerance (IFG, IGT, or IFG and IGT) [42].

### Other measures

To determine the level of physical activity (PA), the Physical Activity Questionnaire-Short Version (IPAQ-SV) was used, which consists of 7 questions that address the frequency, intensity, and duration of PA performed in the last 7 days [43]. Additionally, a survey where data on socioeconomic factors such as marital status, occupation, educational level, sex, health regimen, and cardiovascular history were collected was applied.

### Ethical aspects

This study was approved by the ethics committee of the Universidad del Norte by means of Evaluation Act 141 of April 28, 2016, under the norms of good clinical practice and the guidelines of the Declaration of Helsinki. Each participant was provided with basic information about the study, confidentiality, and data protection; they signed the informed consent before participation and could withdraw from the study whenever they considered it necessary.

### Statistical analysis

Qualitative characteristics of the participants were expressed as absolute numbers and percentages, while quantitative characteristics were expressed as means with standard deviations. The nonparametric distribution of the variables was determined by Kolmogorov–Smirnov or Shapiro–Wilk tests. To evaluate significant differences in quantitative characteristics according to glycemic status, Mann–Whitney and Kruskal–Wallis U tests and the Chi-square test (X^2^) were used for categorical variables. The percentage of problems reported by the participants in the 5 dimensions of quality of life are shown in double bar graphs according to glycemic classification. We examined significant differences in mean EQ-5D and VAS scores among individuals with NGT, prediabetes, and UT2D. Binary logistic regression models by the glycemic group were used to establish statistically significant factors associated with presenting problems in the EQ-5D-3L dimensions. The dependent variable for the logistic regression models was each quality-of-life questionnaire dimension because the percentages of severe or extreme problems (level 3) in the dimensions were very small or null. Thus, we dichotomized the levels of each dimension into "no problems" for level 1 and "some problems" for levels 2 and 3. We then included all variables that had shown statistical significance (< 0.05). However, to summarize the results, only variables that had a statistically significant effect with at least one dimension of quality of life were reported. Finally, to find factors that affected participants' HRQOL, a Tobit regression model was constructed for each glycemic group [44], using the EQ-5D score as the dependent variable. We considered the Tobit regression model appropriate for two reasons. Firstly, the EQ-5D scores are biased and censored at − 0.101 and 1. And secondly a high percentage of the scores (30.6% n = 347) are censored at the upper limit of 1. *P*-values below standard significance (*P*-value < 0.05) were considered statistically significant. Data processing and analysis were performed in R statistical software version 4.0.0.

## Results

### Sample

Data from 1135 participants (76.4% female) were analyzed. Table 1 shows the baseline characteristics of the participants overall and by glycemic classification. The majority were classified as NGT (n = 851, 75.0%), while 16% (n = 182) had prediabetes (IFG, IGT, or IFG and IGT), and 9.0% (n = 102) had undetected type two diabetes (UT2D). Mean age was significantly higher in the prediabetes or UT2D groups (*P*-value < 0.001). Most participants with high glucose levels (Prediabetes or UT2D) were female and from the city of Barranquilla. Participants with UT2D had a higher BMI (31.1 kg/m^2^) compared to NGT (29.6 kg/m^2^). Likewise, mean systolic and diastolic blood pressure was significantly higher in individuals with higher glucose levels. In addition, participants with prediabetes or UT2D had a larger waist circumference than NGTs. The percentage of participants with NGT who received treatment for hypertension was 34.9%, being higher than the percentage presented in participants with prediabetes (51.6%) and UT2D (45.1%). Comparisons between glycemic status and education level, marital status, daily fruit intake, physical activity, and total cholesterol levels were not significant (*P* > 0.05).

**Table 1:** Characteristics of participants according to glycemic status

Characteristics	Total	Glycemic status			*P*-value^b^
		NGT	Prediabetes	UT2D	
	(*n* = 1135)	(*n* = 851)	(*n* = 182)	(*n* = 102)	
Age (years)	55.6 ± 12.1	54.5 ± 12.3	58.7 ± 11.0	59.8 ± 11.2	< 0.001
Gender					
Male	268 (23.6)	184 (21.6)	60 (33.0)	24 (23.5)	0.005^a^
Female	867 (76.4)	667 (78.4)	122 (67.0)	78 (76.5)	
City					
Barranquilla	587 (51.7)	414 (48.6)	110 (60.4)	63 (61.8)	0.002^a^
Bogota, DC	548 (48.3)	437 (51.4)	72 (39.6)	39 (38.2)	
Education level					
No schooling	287 (25.3)	211 (24.8)	41 (22.5)	35 (34.3)	0.188
Elementary school	475 (41.9)	349 (41.0)	88 (48.4)	38 (37.3)	
Junior high school	247 (21.8)	195 (22.9)	33 (18.1)	19 (18.6)	
Superior	125 (11.0)	96 (11.3)	19 (10.4)	10 (9.8)	
Marital status					
Single	198 (17.5)	156 (18.3)	21 (11.6)	21 (20.6)	0.189
Married	706 (62.3)	523 (61.5)	124 (68.5)	59 (57.8)	
Divorced or widower	230 (20.3)	172 (20.2)	36 (19.9)	22 (21.6)	
Daily fruit intake					
No	972 (85.6)	733 (86.1)	156 (85.7)	83 (81.4)	0.432
Physical activity					
Low	854 (75.2)	634 (74.5)	142 (78.0)	78 (76.5)	0.58
Treatment hypertension					
Yes	437 (38.5)	297 (34.9)	94 (51.6)	46 (45.1)	< 0.001
Clinical					
BMI (kg/m^2^)	30.0 ± 4.8	29.6 ± 4.6	30.8 ± 5.2	31.1 ± 5.4	< 0.001
Systolic blood pressure (mmHg)	127.9 ± 24.5	125.6 ± 24.4	135.9 ± 23.3	132. ± 24.4	< 0.001
Diastolic blood pressure (mmHg)	84.1 ± 13.9	83.4 ± 14.0	86.8 ± 13.0	84.5 ± 14.1	0.002^a^
Waist circumference (cm)	100.0 ± 10.4	99.0 ± 10.0	102.9 ± 11.3	103.3 ± 10.1	< 0.001
Total cholesterol (mg/dl)	202.5 ± 44.5	203.3 ± 45.1	199.8 ± 43.3	205.4 ± 46.9	0.728
HDL cholesterol (mg/dl)	47.6 ± 10.4	48.1 ± 10.7	46.5 ± 9.3	44.9 ± 9.2	0.013^a^

Mean ± SD or n (%)

BMI, body mass index; HDL, high-density lipoprotein; NGT: patients with normoglycemic, Prediabetes: Patients with impaired fasting glucose (IFG), impaired glucose tolerance (IGT) or IFG and IGT; UT2D: Patients with unknow diabetes

^a^Comparisons were significant (*P*-value < 0.01)

^b^*P*-values are based on Kruskal–Wallis test for continuous and Chi square test for categorical variables

### EQ-5D-3L results

**Fig. 1:**
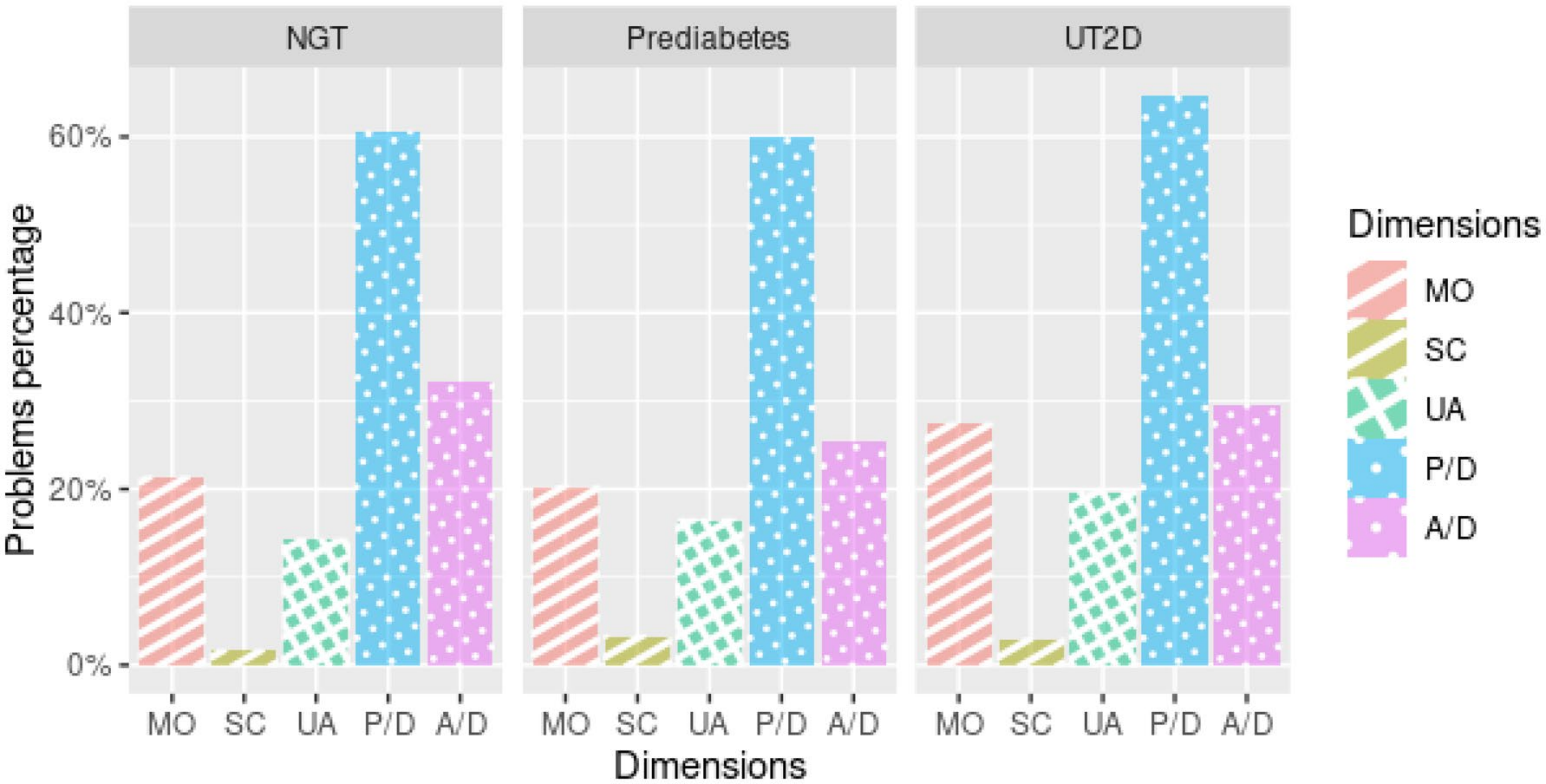
Percentage of problems reported by participants in terms of glycemic status. Percentage of each level of problems in 5 dimensions of the HRQOL reported by glycemic status; NGT: patients with normoglycemic, Prediabetes: Patients with impaired fasting glucose (IFG), impaired glucose tolerance (IGT) or IFG and IGT; UT2D: Patients with unknow diabetes; MO; Mobility; SC: Self-Care; UA; Usual Activities; P/D: Pain/Discomfort and A/D: Anxiety/Depression

The percentages of moderate or severe problems reported by participants in each glycemic group in the five dimensions of the EQ-5D-3L description system are shown in Fig. 1. Most participants reported having no problems in several dimensions of the quality-of-life questionnaire. The dimensions with the least reported problems were self-care (NGT = 1.6%; prediabetes = 3.3%; and UT2D = 2.9%) and usual daily activities (NGT = 14.2%; prediabetes = 16.5%; and UT2D = 19.6%). However, at a high percentage the participants in this study reported some problem (moderate or severe) in the dimensions of mobility (NGT = 21.3%; prediabetes = 20.3%; and UT2D = 27.4%), pain/discomfort (NGT = 60.5%; prediabetes = 59.9%, and UT2D = 64.7%), and anxiety/depression (NGT = 32.2%; prediabetes = 25.3%; and UT2D = 29.4%). The percentage of problems in each of the dimensions did not differ significantly when compared by glycemic status.

Table 2 shows the mean scores of the EQ-5D-3L and EQ-VAS according to demographic and clinical characteristics of the participants by glycemic status. Mean utility scores were statistically similar in the NGT (0.80; 95% CI 0.79–0.81), prediabetes (0.81; 95% CI 0.79–0.83), and those with UT2D (0.79; 95% CI 0.76–0.82) groups. Likewise, mean EQ-VAS scores did not differ significantly by glycemic status. However, mean EQ-5D and EQ-VAS scores were significantly lower in NGT (0.79 vs. 0.83 for EQ-5D and 74.3 vs. 77.4 for VAS score) and prediabetic (0.78 vs. 0.86 for EQ-5D and 72.1 vs. 78.3 for VAS score) women compared with men in the same glycemic group. Participants older than 64 years with NGT had significantly lower EQ-5D and EQ-VAS scores compared to younger NGTs. In terms of city, mean HRQOL scores were lower in Bogotá residents in each of the glycemic groups. Divorced or widowed NGTs or UT2D reported statistically lower EQ-5D and VAS scores compared to married people in the same glycemic group. The EQ-5D and EQ-VAS scores of those NGT participants receiving treatment for hypertension were statistically lower compared to those who reported no hypertension problems. However, mean EQ scores across the different glycemic groups were similar in terms of participant BMI classification.

**Table 2:** Comparison of mean EQ-5D score and EQ-VAS according to patient demographic characteristics by glycemic status

Characteristics	EQ-5D score			EQ-VAS		
	NGT	Prediabetes	UT2D	NGT	Prediabetes	UT2D
	Mean (CI 95%)	Mean (CI 95%)	Mean (CI 95%)	Mean (CI 95%)	Mean (CI 95%)	Mean (CI 95%)
Total	0.80 (0.79–0.81)	0.81 (0.79–0.83)	0.79 (0.76–0.82)	75.0 (73.8–76.1)	74.1 (71.5–76.7)	69.5 (65.5–73.6)
Sex						
Male	0.83 (0.80–0.85)	0.86 (0.82–0.90)	0.75 (0.70–0.80)	77.4 (74.8–79.9)	78.3 (74.6–82.0)	70.8 (60.2–81.4)
Female	0.79 (0.78–0.80)	0.78 (0.75–0.81)	0.80 (0.77–0.84)	74.3 (73.0–79.9)	72.1 (68.7–75.4)	69.1 (64.8–73.5)
*P*-value	0.001*	0.002*	0.131	0.007*	0.045*	0.354
Age group (years)						
30–45	0.83 (0.81–0.85)	0.83 (0.78–0.88)	0.87 (0.78–0.95)	78.5 (76.6–80.4)	78.6 (69.5–87.7)	71.8 (60.3–83.4)
45–54	0.80 (0.78–0.82)	0.84 (0.80–0.88)	0.80 (0.73–0.86)	74.8 (72.7–77.0)	75.7 (70.5–80.9)	72.4 (62.6–82.2)
55–64	0.78 (0.76–0.80)	0.81 (0.77–0.85)	0.79 (0.74–0.84)	74.5 (72.4–76.6)	73.6 (69.0–78.2)	68.7 (61.8–75.7)
> 64	0.77 (0.75–0.80)	0.77 (0.72–0.83)	0.77 (0.72–0.82)	72.1 (69.3–74.8)	71.8 (67.2–76.3)	67.8 (60.3–75.2)
*P*-value^‡^	0.001*	0.103	0.278	0.007*	0.187	0.66
City						
Barranquilla	0.85 (0.84–0.87)	0.87 (0.84–0.89)	0.83 (0.80–0.86)	76.6 (75.1–78.1)	75.6 (72.5–78.6)	72.9 (68.8–77.0)
Bogota, DC	0.74 (0.73–0.76)	0.72 (0.68–0.76)	0.72 (0.67–0.78)	73.4 (71.7–75.0)	71.9 (67.3–76.6)	64.1 (55.7–72.4)
*P*-value^†^	< 0.001**	< 0.001**	< 0.001**	0.012*	0.291	0.17
Marital status						
Single	0.78 (0.75–0.80)	0.82 (0.74–0.89)	0.83 (0.76–0.91)	73.2 (70.5–75.8)	73.7 (64.8–82.7)	74.3 (66.7–81.8)
Married	0.81 (0.80–0.82)	0.82 (0.79–0.85)	0.79 (0.75–0.82)	75.7 (74.3–77.1)	74.9 (71.9–77.8)	72.8 (68.2–77.4)
Divorced or widower	0.77 (0.74–0.79)	0.76 (0.71–0.82)	0.76 (0.70–0.82)	74.3 (71.8–76.8)	73.6 (67.6–79.6)	56.3 (44.6–67.9)
*P*-value^‡^	0.001*	0.114	0.202	0.124	0.991	0.017*
Hypertension						
No	0.80 (0.79–0.82)	0.83 (0.80–0.86)	0.78 (0.74–0.82)	76.1 (74.7–77.5)	74.5 (70.2–78.8)	72.6 (67.3–77.8)
Yes	0.78 (0.76–0.80)	0.79 (0.76–0.83)	0.81 (0.76–0.85)	72.8 (70.9–74.8)	73.8 (70.7–76.8)	65.8 (59.5–72.2)
*P*-value^†^	0.045*	0.121	0.278	0.003*	0.241	0.092
BMI (kg/m^2^)						
Normal weight	0.79 (0.76–0.83)	0.84 (0.77–0.91)	0.78 (0.64–0.93)	73.7 (70.7–76.8)	74.4 (66.6–82.3)	62.2 (40.9–83.5)
Overweight	0.80 (0.78–0.81)	0.81 (0.76–0.85)	0.77 (0.72–0.82)	75.4 (73.7–77.1)	71.1 (65.4–76.7)	69.4 (62.0–76.8)
Obesity	0.79 (0.78–0.81)	0.80 (0.77–0.84)	0.81 (0.77–0.84)	74.9 (73.1–76.6)	75.9 (73.1–78.8)	70.8 (65.8–75.7)
*P*-value^‡^	0.756	0.785	0.344	0.517	0.686	0.786

BMI, body mass index; NGT: patients with normoglycemic, Prediabetes: Patients with impaired fasting glucose (IFG), impaired glucose tolerance (IGT) or IFG and IGT; UT2D: Patients with unknow diabetes. *P*-value: ^†^*Mann–Whitney U test; ^‡^Kruskal–Wallis test. No marks: not significant; *P < 0.05; **P < 0.01; CI, confidence interval; EQ-VAS, visual analog scale

### Factors associated with HRQOL

Table 3 shows the results of the logistic regression models by glycemic group for each of the five dimensions of the EQ-5D-3L. The factors that were significantly associated with a higher report of problems in some dimension of quality of life in participants with NGT were the largest city, oldest age, female sex, hypertension, education, and low physical activity. In prediabetes, they were largest city, oldest age, female sex, and low physical activity; while, in those with UT2D, only largest city and female sex were significantly associated. In the prediabetes group, larger city (OR 2.3; 95% CI 1.2–4.5) and female sex (OR 2.4; 95% CI 1.3–4.6) were significantly associated with higher report of problems in the P/D dimension. In the UT2D group, larger city (OR 5.2; 95% CI 1.9–13.8) and older age (OR 5.3; 95% CI 1.4–19.9) were significantly associated with higher report of problems in the MO dimension; likewise, larger city was associated with the UA and A/D dimensions. In the SC dimension, no significant associations were found in the prediabetes and UT2D groups. Likewise, no significant associations were found in participants with UT2D in P/D.

**Table 3:** Factors associated with presenting problems in the of quality-of-life dimensions for glycemic group in Barranquilla and Bogota, DC

Dependent variable	Characteristic	Glycemic group		
		NGT	Prediabetes	UT2D
		(*n* = 851)	(*n* = 182)	(*n* = 102)
		OR (95% CI)	OR (95% CI)	OR (95% CI)
Mobility	City			
	Barranquilla (Ref.)			
	Bogota, DC	5.5 (3.7–8.4)	9.1 (3.6–22.9)	5.2 (1.9–13.8)
	Age group			
	< 55 years (Ref.)			
	≥ 55 years	2.1 (1.4–3.1)	10.2 (2.3–45.9)	5.3 (1.4–19.9)
	Sex			
	Male (Ref.)			
	Female	1.7 (1.05–2.6)		
	Hypertension treatment			
	No (Ref.)			
	Yes	1.7 (1.2–2.4)		
Self-care	City			
	Barranquilla (Ref.)			
	Bogota, DC	5.8 (1.3–26.1)	–	–
Usual activities	City			
	Barranquilla (Ref.)			
	Bogota, DC	9.8 (5.3–18.0)	19.1 (5.4–66.8)	10.3 (3.1–34.0)
	Age group			
	< 55 years (Ref.)			
	≥ 55 years	1.8 (1.1–3.0)	4.2 (1.1–15.8)	
	Sex			
	Male (Ref.)			
	Female	2.1 (1.2–3.8)		
	Treatment Hypertension			
	No (Ref.)			
	Yes	1.8 (1.1–2.7)		
	Education level			
	Superior (Ref.)			
	No schooling	0.72 (0.34–1.5)		
	Elementary school	0.45 (0.22–0.94)		
	Junior high school	0.29 (0.12–0.71)		
	Physical activity			
	Moderate or High (Ref)			
	Low	1.8 (1.1–2.9)		
Pain/discomfort	City			
	Barranquilla (Ref.)			
	Bogota, DC	1.7 (1.2–2.3)	2.3 (1.2–4.5)	–
	Sex			
	Male (Ref.)			
	Female	1.7 (1.2–2.4)	2.4 (1.3–4.6)	–
	Physical activity			
	Moderate or High (Ref)			
	Low	1.5 (1.03–2.1)		
Anxiety/depression	City			
	Barranquilla (Ref.)			
	Bogota, DC	8.5 (6.0–12.2)	22.8 (8.4–61.4)	7.0 (2.7–18.0)
	Sex			
	Male (Ref.)			
	Female	1.7 (1.1–2.6)	4.2 (1.5–11.4)	
	Physical activity			
	Moderate or high (Ref)			
	Low		2.9 (1.06–7.7)	

NGT: patients with normoglycemic, Prediabetes: Patients with impaired fasting glucose (IFG), impaired glucose tolerance (IGT) or IFG and IGT; UT2D: Patients with unknow diabetes. The dependent variable for the logistic regression models was each quality-of-life questionnaire dimension. OR, Odds Ratio; Ref.: reference group

The results of the Tobit regression models for each of the glycemic states, like the model for the total population, are shown in Table 4. The variables for the EQ-5D score in the total population showed statistical significance in women), in participants who were aged 45–54 years, 55–64 years, older than 64 years, and for those living in the smallest city. The results of the Tobit regression model for the NGT group showed significant differences in quality of life in women, in participants aged 45–54 years, those aged 55–64 years, those living in the smallest city, and participants with primary education. In prediabetes, the significant results were in women, in those living in the smallest city, and in participants receiving treatment for hypertension), while, in UT2D, only city and marital status yielded significant results.

**Table 4:** Results of Tobit regression model for total and glycemic classification

Variable	Total	NGT	Prediabetes	UT2D
	(*n* = 1135)	(*n* = 851)	(*n* = 182)	(*n* = 102)
	β (SE)^†^	β (SE)^†^	β (SE)^†^	β (SE)^†^
Gender				
Male	Ref.	Ref.	Ref.	Ref.
Female	− 0.06 (0.015)*	− 0.05 (0.018)*	− 0.12 (0.036)*	0.03 (0.044)
Age group (years)				
30–45	Ref.	Ref.	Ref.	Ref.
45–54	− 0.04 (0.019)*	− 0.05 (0.021)*	0.03 (0.058)	− 0.06 (0.069)
55–64	− 0.05 (0.020)*	− 0.05 (0.022)*	0.00 (0.058)	− 0.05 (0.068)
> 64	− 0.05 (0.022)*	− 0.05 (0.025)	0.04 (0.064)	− 0.08 (0.069)
City				
Bogotá, DC	Ref.	Ref.	Ref.	Ref.
Barranquilla	0.14 (0.012)*	0.14 (0.015)*	0.18 (0.035)*	0.12 (0.040)*
Education level				
No schooling	Ref.	Ref.	Ref.	Ref.
Elementary school	0.03 (0.016)	0.04 (0.019)*	0.03 (0.045)	0.01 (0.042)
Junior high school	0.01 (0.020)	0.02 (0.023)	0.01 (0.055)	− 0.02 (0.055)
Superior	0.01 (0.024)	0.02 (0.028)	0.04 (0.067)	− 0.04 (0.068)
Marital status				
Single	Ref.	Ref.	Ref.	Ref.
Married	− 0.02 (0.020)	− 0.01 (0.023)	− 0.07 (0.058)	− 0.13 (0.057)*
Divorced or widower	0.00 (0.017)	0.02 (0.019)	− 0.04 (0.051)	− 0.10 (0.049)*
BMI (kg/m^2^)				
Normal weight	Ref.	Ref.	Ref.	Ref.
Overweight	− 0.01 (0.020)	− 0.00 (0.023)	− 0.05 (0.061)	− 0.05 (0.070)
Obesity	− 0.02 (0.020)	− 0.02 (0.023)	− 0.04 (0.058)	− 0.01 (0.071)
Hypertension treatment				
No	Ref.	Ref.	Ref.	Ref.
Yes	− 0.02 (0.014)	− 0.01 (0.016)	− 0.07 (0.034)*	0.07 (0.040)
Family history of diabetes				
No	Ref.	Ref.	Ref.	Ref.
Yes, second degree	− 0.01 (0.018)	− 0.02 (0.021)	0.03 (0.048)	0.08 (0.058)
Yes, first degree	− 0.02 (0.016)	− 0.02 (0.018)	− 0.05 (0.041)	0.08 (0.044)

BMI, body mass index; NGT: patients with normoglycemic, Prediabetes: Patients with impaired fasting glucose (IFG), impaired glucose tolerance (IGT) or IFG and IGT; UT2D: Patients with unknow diabetes, ^†^Tobit model regression coefficients (β); SE, Standard error; *Statistically signifcant *P*-value < 0.05

## Discussion

Our data indicate that gender, age, place of residence, educational level, marital status, and hypertension treatment were associated with HRQOL when compared by glycemic group. Likewise, we found that the greatest report of problems in each glycemic group was presented in the dimensions of Pain/Discomfort and Anxiety/Depression. The most frequent alterations in the dimensions of quality of life and the lowest HRQOL scores in the different glycemic groups were associated to a greater extent with female participants, residents of the larger city (Bogotá) and with older participants.

To our knowledge, this is one of the few studies that establishes the HRQOL of participant at risk for diabetes by means of the FINDRISC instrument, which includes participants with NGT, prediabetes, and UT2D, using the EQ-5D. Most studies of HRQOL in population at risk for diabetes do not include participants with NGT or have assessed HRQOL with other questionnaires such as the SF-36 [19, 20, 23], SF-6D [23], and the 15D HRQoL [22]. Likewise, studies assessing HRQOL in patients with UT2D are scarce [45]. Recent scientific literature revealed that the EQ-5D has been widely used to measure HRQOL in diabetic patients in different countries [8, 13, 16, 18, 31], which is probably due to the ease of use of this instrument in the use of health economic evaluations, hence our interest in establishing the HRQOL of our participants with this questionnaire.

Our findings show that the dimensions of quality of life in participants with NGT, prediabetes, and UT2D with the highest reported problems are Pain/Discomfort and Anxiety/Depression, being like the results found in population at risk for diabetes [24] and consistent with studies that measured HRQOL in patients with diabetes [8–10], and, partially, with those involving patients with prediabetes or NGT [11, 12, 19, 22]. Previous studies in patients with type 2 diabetes have reported that the dimensions with the highest reported problems are Pain/Discomfort and Anxiety/Depression [8–10], however, Sakamaki et al., in a 2006 study of diabetic patients in Japan reported greater problems in the dimension’s Pain/Discomfort and Mobility [13].

Regarding people with prediabetes, there is no clear consensus, either due to the use of different methods of measuring HRQOL or to the scarce and controversial literature in this type of population. For example, while some studies in patients with prediabetes showed that the problems most likely reported were the dimensions of physical functioning and bodily pain [11, 12, 19], the study by Seppälä et al. reported that prediabetes was not associated with low HRQOL [20]. However, a study in Greece reported that mobility and psychological distress are the dimensions with the greatest impairment in patients with prediabetes (IGT) [22]. In addition, Adriaanse MC et al. observed that depressive symptoms were higher in women with prediabetes compared to participants with normal glucose metabolism [46], however, a meta-analysis in 2011 concluded that patients with normoglycemia, prediabetes, and undetected type two diabetes have similar risk of depression [47].

In the present study, the mean EQ-5D score in participants with NGT, prediabetes, and UT2D was 0.80, 0.81, and 0.79 respectively, whereas the VAS scores in NGT, prediabetes, and UT2D were 75.0, 74.1, and 69.5, different from those reported in a recent study in Denmark where the mean EQ-5D scores in NGT, prediabetes, and UT2D were 0.90, 0.86, and 0.85 [45]. In the study of Abedini et al. with diabetic patients in Iran, the mean EQ-5D and VAS scores were 0.89 and 65.22, respectively [8]. Studies with diabetic patients in Indonesia and India showed similar results to ours, the mean EQ-5D scores were 0.77 and 0.803 respectively [16, 17]. Similar studies with diabetic patients using the EQ-5D in Iran, Korea, Japan, and the Netherlands reported mean EQ-5D and VAS scores of 0.70 and 56.8, 0.87 and 71.94, 0.86 and 74.3, 0.74 and 68.0, respectively [9, 10, 13, 15]. Other studies reported that the mean EQ-5D score in diabetic patients was 0.85 [48] and 0.75 [14].

On the other hand, Makrilakis et al. in 2018 in a study in Greece, using the 15D HRQOL questionnaire reported scores of 0.91, 0.90, and 0.86 for patients with NGT, prediabetes, and T2D each [22]. Because various factors influence the HRQOL of individuals, the HRQOL scores of this study with those found in diabetic, prediabetic, or NGT population should be compared and interpreted with caution. These differences could be related to differences in the main characteristics of the subjects or to different methods of measuring HRQOL (the set of EQ-5D values used by each country is different). On the other hand, some of these studies worked with patients who were undergoing treatment for diabetes or already had complications due to the disease, whereas the participants in our study were administered the Quality-of-Life questionnaire before establishing their glycemic status, i.e. they were unaware of their diagnosis of prediabetes or occult diabetes. In our study, overall, the HRQOL of NGT, prediabetes, and UT2D were significantly equal, however, when examining certain characteristics within each glycemic group, significant differences were found in the EQ-5D score.

Our study showed that mean EQ-5D scores in participants with NGT or prediabetes were lower in females as compared to males, being similar and consistent with studies in diabetic population using the same questionnaire [8, 9, 14]. Along the same lines, Neumann et al. reported that male sex was associated with a higher quality of life score [23]. In contrast, Makrilakis et al., in 2018, reported that male sex was significantly associated with lower HRQOL [22]. The results of our study showed that older age was associated with lower HRQOL in NGT participants like that reported in another study [23]. The HRQOL scores of our UT2D participants were similar across age groups. In contrast to our results, other studies with diabetic patients report that older age groups were associated with lower HRQOL [10, 12, 15], however, O'Reilly et al., in their study, report that the EQ-5D score of diabetics increases with age [14]. These differences could be due to the lack of knowledge about diabetes or the characteristics of our participants. Another significant finding in the present study is that participants with NGT, prediabetes, or UT2D who resided in the larger city had lower HRQOL scores compared to those who resided in the smaller city, being consistent with the results reported by Javanbakht et al. in diabetic patients [15].

Our results showed that larger city, older age, female sex, education, being on medication for hypertension, and low physical activity are associated with higher odds of reporting problems in some dimension of the EQ-5D. Reporting problems on the MO dimension are more likely in participants residing in the largest city and having the oldest age, similar with the results found in patients with T2D [8, 10, 15]. Participants with NGT or prediabetics who are female and reside in the largest city are more likely to report problems in the P/D and A/D dimensions, likewise, participants with UT2D who reside in the largest city are more likely to report problems in the A/D dimension, partially correlating with the results of Abedini MR et al. in a diabetic population [8]. Participants with NGT living in larger cities are more likely to report problems in the SC dimension just like the results of Javanbakht M, but in patients with T2D [15]. The results of the Tobit regression model showed that female sex, older age, living in the largest city, being on hypertension medication, marital status and lower education were significantly associated with lower EQ-5D scores. Javanbakht et al. demonstrated, using a similar model in diabetic patients, that female sex, living in the largest city, and lower education were significantly associated with lower EQ-5D scores [15].

The results of this study are important because they provide utility values by glycemic status for this population in Colombia, facilitating the calculation of quality-adjusted life years (QALYs), essential for health economic evaluations. However, future studies should replicate this study to validate utility values in this type of population, to determine whether self-reported perceptions of HRQOL by glycemic status are consistent with the participants' level of utility. Naturally, our study has some limitations related to the fact that the examined population is not necessarily representative of the general population, since the participants in this study are from lower socioeconomic strata; therefore, the findings are not necessarily applicable to the general population. Another limitation of this study is not having a set of EQ-5D-3L values adjusted for Colombia, so it was necessary to use the set of Latin American values [39], assuming some similarity in the Latin American context at the population level, language, and cultural habits/customs. Likewise, the ceiling effect of the EQ-5D-3L and the low sensitivity to detect small changes in HRQOL compared with the 5-level version (EQ-5D-5L) [49] could be considered another limitation; however, we preferred to use the 3-level version because it is the version most recommended by health economic evaluation agencies for the calculation of QALYs [40, 41, 49]. We also did not include potentially useful variables such as participants' current treatments or comorbidities. Finally, as this was a cross-sectional study, the associations observed are not necessarily causal.

## Conclusions

The findings found in this study indicate that the HRQOL of people at risk of diabetes did not differ significantly by glycemic group. However, factors such as gender, age, place of residence, educational level, marital status, and receiving medications for hypertension were significant factors for HRQOL. Due to the above, these variables/factors identified as effect modifiers on HRQOL of the different glycemic groups; could become crucial determinants to design, reorient and implement strategies to identify subjects at risk of diabetes or with unknown glycemic alteration and aim to obtain better health outcomes within primary care institutions. However, future studies that can contrast or validate these results are expected; and it is invited that they should focus on establishing comparisons of HRQOL in the general population with those at risk of diabetes and evaluating the impact of strategies on changes in HRQOL in each glycemic group. This would provide more rigorous evidence to be able to make decisions in health and to be able to design a territorial public policy strategy adjusted to the population's needs that impacts the prevention of diabetes mellitus in the medium term and, in turn, affects the strengthening and prevention of the deterioration of the HRQOL of the groups with early glycemia alterations.

## Data Availability

All authors certify that they have no affiliations with or involvement in any organization or entity with any financial or nonfinancial interest in the subject matter or materials discussed in this manuscript. The datasets presented in this article are not readily available because The PREDICOL Project is a Project that is currently in progress, so the database according to the regulations may not be published until the end of the study. For any requirement you can contact the authors. Requests for access to the datasets should be directed to laanillo@uninorte.edu.co.

## Abbreviations

HRQOL: Health-related quality of life
T2D: Type 2 diabetes
OGTT: Oral glucose tolerance test
IGT: Impaired glucose tolerance
IFG: Impaired fasting glycemia
UT2D: Diabetics do not know they have diabetes
NGT: Normoglycemic subjects
VAS: Visual analog scale
FINDRISC: The Finnish Diabetes Risk Score
SF-36: Short form 36
HUI: The health utility index
PROs: Patient-reported outcome measures
MO: Mobility
SC: Self-care
UA: Usual activities
P/D: Pain/discomfort
A/D: Anxiety/depression
BMI: Body Mass Index
PA: Physical activity
IPAQ-SV: The Physical Activity Questionnaire-Short Version
FPG: Fasting plasma glucose
2hPG: Plasma glucose at 2 h
